# Prenatal Sonographic Image of Sirenomelia with Anencephaly and Craniorachischisis Totalis

**DOI:** 10.1155/2018/7058253

**Published:** 2018-11-28

**Authors:** Takako Sugiura, Yuka Sato, Naoyuki Nakanami, Kiyomi Tsukimori

**Affiliations:** Department of Obstetrics, Perinatal Center, Fukuoka Children's Hospital, 5-1-1 Kashiiteriha, Higashi-ku, Fukuoka 813-0017, Japan

## Abstract

Sirenomelia is a rare congenital malformation characterized by varying degrees of fusion of the lower extremities. It is commonly associated with severe urogenital and gastrointestinal malformations; however, the association of sirenomelia with anencephaly and rachischisis totalis is extremely rare. To our knowledge, the prenatal sonographic images of this association have not been previously published. Here, we present prenatal sonographic images of this association, detected during the 17th week of gestation through combined two-dimensional, four-dimensional, and color Doppler ultrasound. Two-dimensional ultrasound images showed anencephaly, spina bifida, and possible fusion of the lower limbs. Three-dimensional HDlive rendering images confirmed the final diagnosis of sirenomelia with anencephaly and rachischisis totalis. The patient opted to undergo medical termination of pregnancy and delivered a fetus with fused lower limbs, anencephaly, and rachischisis totalis confirming the* in utero* imaging findings. Awareness of these rare associations will help avoid misdiagnoses and facilitate prenatal counselling. This case highlights the importance of a thorough ultrasound examination.

## 1. Introduction

Sirenomelia is a rare congenital malformation characterized by varying degrees of fusion of the lower extremities in association with lumbosacral and pelvic bone abnormalities, absent external genitalia, anorectal atresia, single umbilical artery, and renal agenesis [1]. The association of sirenomelia with anencephaly and rachischisis totalis is extremely rare; only eight cases have been reported in the literature to date [2–4]. Here, we present a case of sirenomelia associated with anencephaly and rachischisis totalis detected during the 17th week of gestation through combined two-dimensional (2D), four-dimensional (4D), and color Doppler ultrasound. To our knowledge, prenatal sonographic images of this association have not been published previously.

## 2. Case Presentation

**Figure 1:**
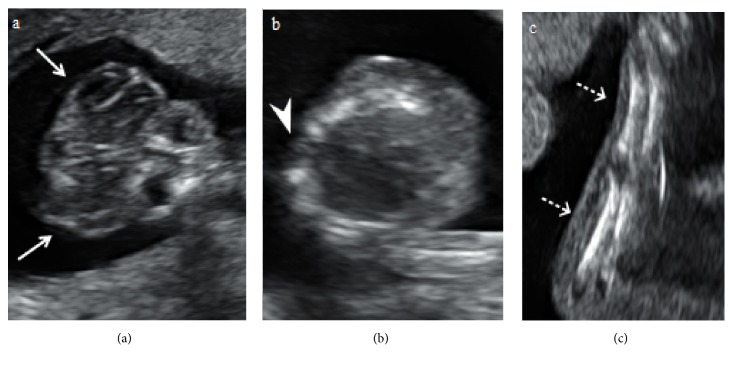
Two-dimensional ultrasound images at 17 weeks of gestation. (1a) The fetus demonstrating typical features of anencephaly (“Mickey Mouse” sign; white arrows). The calvarium is absent, and the brain is “floating” in the amniotic fluid. (1b) Absence of vertebral posterior elements with splaying of the lamina at the thoracic level (white arrowhead). (1c) The lower limbs appeared to be fused together in fixed extension with two femora and two tibiae (white dashed arrows).

**Figure 2:**
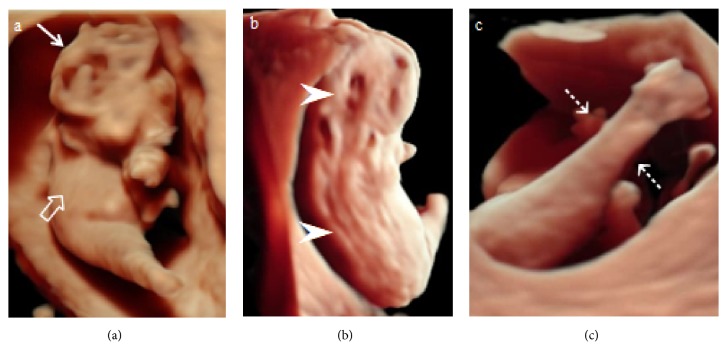
Three-dimensional HDlive rendering images at 17 weeks of gestation. (2a) Deformed and degenerated brain tissues are evident (white arrow). Absence of the right upper limb (white open arrow). (2b) The spine showed extensive clefts in the posterior part of the fetal vertebrae from the upper cervical region to the sacrum (white arrowheads). (2c) The lower extremities were completely fused, and the feet were fused with the heels, which were immobile (white dashed arrows).

A 28-year-old primigravida woman was referred to our hospital for the evaluation of a suspected fetal cranial abnormality at 17 weeks of gestation. There was an unremarkable medical history and family history and no history of drugs or substance abuse. 2D ultrasound images (Voluson E8; GE Medical Systems, Zipf, Austria) revealed the absence of calvarium with deformed brain tissue directly exposed to the amniotic cavity, suggestive of exencephaly (Figure 1(a)). The fetal spine also showed the absence of vertebral posterior elements with splaying of the lamina at the thoracic level (Figure 1(b)). The lower limbs appeared to be fused in fixed extension with two femora and two tibiae (Figure 1(c)); however, we were not able to identify whether there were one or two feet. The fetal kidneys and urinary bladder were visualized as normal, and the amniotic fluid was normal. Color Doppler showed a single umbilical artery. 3D rendering images using 4D ultrasound with HDlive mode clearly revealed the absence of calvarium with deformed and degenerated brain tissues, consistent with exencephaly (Figure 2(a)). The spine also showed extensive clefts in the posterior part of the fetal vertebrae from the upper cervical region to the sacrum (Figure 2(b)). These findings were consistent with those of craniospinal rachischisis totalis (anencephaly with rachischisis totalis). The lower extremities were completely fused, and the feet were fused with the heels, which were immobile (Figure 2(c)). Absence of the right upper limb was also identified (Figure 2(a)). Thus, the diagnosis of sirenomelia with anencephaly, rachischisis totalis, and absence of the right upper limb was made prenatally.

**Figure 3:**
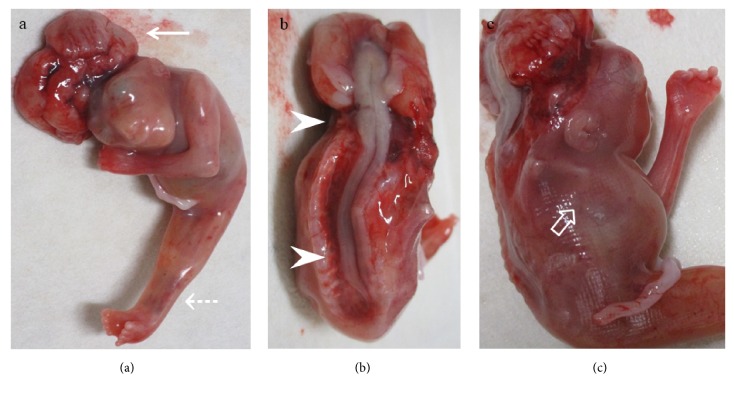
Macroscopic views of the aborted fetus. (3a) External examination of the fetus showed anencephaly (white arrow), fused lower limbs, and nine toes with a fused bilateral thumb (white dashed arrows). (3b) Craniorachischisis totalis (white arrowheads). (3c) Absence of the right upper limb (white open arrow).

**Figure 4:**
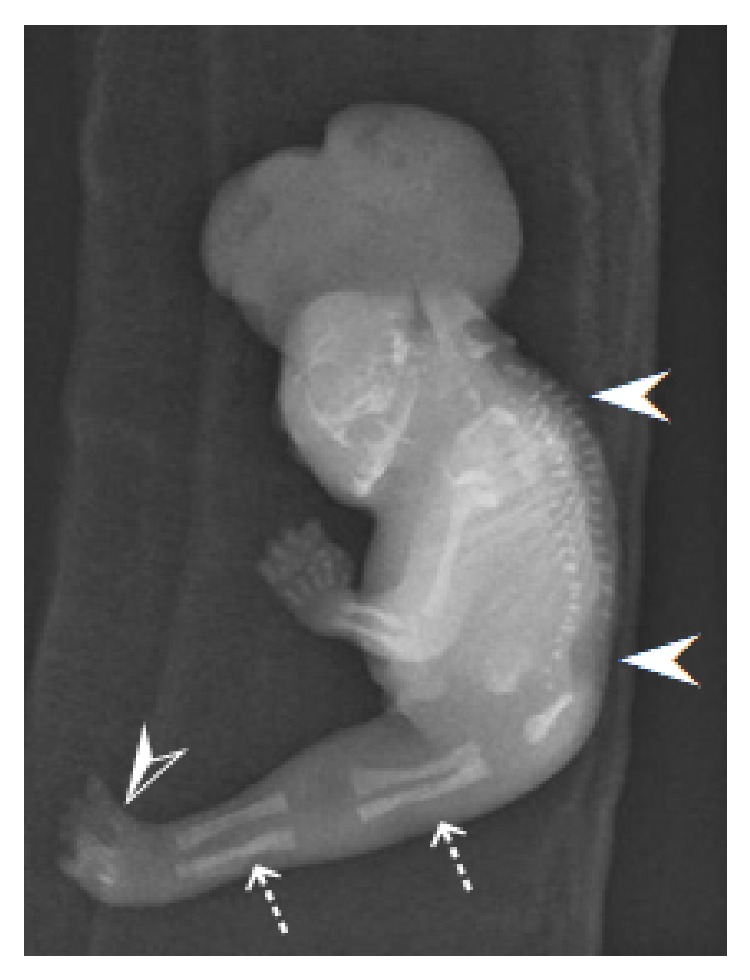
Autopsy imaging by radiography. The radiographs demonstrated complete rachischisis (white arrowheads) and the single lower limb containing two femora and two tibiae (white dashed arrows) and some metatarsals and phalanges (white open arrowhead).

The parents were informed about the findings and the associated poor prognosis; they then opted for termination of pregnancy, which was performed at 18 weeks of gestation. External examination of the fetus revealed anencephaly with craniorachischisis totalis (Figure 3(a)), fused lower limbs (Figure 3(a)), nine toes with a fused bilateral thumb (Figure 3(a)), absence of the right upper limb (Figure 3(c)) and external genitalia, and imperforate anus, almost coinciding with the observation on the 3D sonographic rendering images. The autopsy imaging by radiography demonstrated complete rachischisis (Figure 4). The single lower limb contained two femora and two tibiae with some metatarsals and phalanges (Figure 4).

## 3. Discussion

Sirenomelia sequence is a very rare congenital malformation. The incidence of sirenomelia varies between 1.1 and 4.2 per 100,000 births and does not differ among ethnic groups [5]. The association of sirenomelia with anencephaly and rachischisis totalis is an extremely rare condition; to our knowledge, only eight cases of this association have been reported in the literature [2–4]. Previous reports of sirenomelia with anencephaly and rachischisis totalis in the fetus were retrospective diagnoses. Theofanakis et al. [4] described a case of anencephaly, spina bifida, and possible single femoral bone that could not confirm the diagnosis of either sirenomelia or rachischisis totalis prenatally. Therefore, our case report is the first to describe prenatal sonographic images of sirenomelia with anencephaly and rachischisis totalis.

Anencephaly can be easily detected by 2D ultrasound; the characteristic appearances are the “Mickey Mouse” sign in early pregnancy and the “frog eyes” sign in the second trimester [6]. In contrast, prenatal sonographic detection of the lower limb anomalies associated with sirenomelia, especially the single or fused lower limbs, is often limited by the oligohydramnios that occurs as a result of the bilateral renal agenesis [7]. Several authors have reported cases of sirenomelia diagnosed in the first trimester, as early as 9 weeks of gestation, using 2D, 3D, and color Doppler ultrasound [8–11]. These findings include single or fused lower extremities, a single umbilical artery contiguous with the aorta, absent urinary bladder, absent kidney, and intra-abdominal cystic structures. In our case, on 2D ultrasound, the amniotic fluid volume was normal, so we could observe the “Mickey Mouse” exencephaly appearance. The lower limbs appeared to be fused together in fixed extension with two femora and two tibiae; however, we were not able to identify whether there were one or two feet. The 3D HDlive rendering images clearly depicted the fused lower limbs, confirmed by macroscopic evaluation of the fetus. Moreover, with a detailed study using the 3D HDlive rendering images, we found other pathological features in this fetus, including rachischisis totalis and absence of the right upper limb.

HDlive is a new surface 3D render mode. The system uses an adjustable light source, affording the operator the opportunity to create light and shadow effects, thereby increasing depth perception [12]. With respect to the major advantages of HDlive, its pictures of embryos and fetuses are more readily discernible than those obtained by conventional 3D ultrasound [13]. In the fetus with sirenomelia, HDlive has been shown to allow acquisition of extraordinary image quality, including single femur, fused lower limbs, and a single inverted foot with oligodactyly [14]. Moreover, HDlive provides entirely new visual experiences for couples and their families, owing to the anatomically realistic depiction of normal fetal development and fetal anomalies [13, 15, 16]. Therefore, in this case, we used the 4D ultrasound with HDlive mode for evaluation of the fetus, so we could easily observe the fused lower limbs, confirming the prenatal diagnosis of sirenomelia. The 3D HDlive rendering mode has the potential to supplement conventional 2D and 3D ultrasound in diagnosing fetal anomalies and to provide detailed information about anatomical characteristics of the fetus.

The etiology of sirenomelia syndrome remains unknown. No instances of familial recurrence of sirenomelia have been reported [17]. Almost all of the karyotypes performed on sirenomelia fetuses reported in the literature were normal. Recently, a case of a triploid mosaic (69, XXX/46, XX) fetus with sirenomelia has been reported [18]. Another case of sirenomelia with a reciprocal translocation 46X, t(X;16) (p11.23;p12.3) has also been reported; however, the chromosomal breakpoints on the pairs of chromosomes did not disrupt the coding genes associated with early human development, especially with blastogenesis [19]. In animal models (mice) with sirenomelia, mutations in the superfamily of cytochrome P450 (CYP) genes, specifically CYP26A1, an enzyme that degrades retinoic acid, have been noted [5]. Another observation is the link between bone morphogenic protein 7 (BMP7) and twisted gastrulation (Tsg); loss of BMP7 combined with a complete loss or half-dose of Tsg in mice models was associated with sirenomelia [20]. These studies have not been replicated in humans. Therefore, the molecular mechanisms producing sirenomelia remain undetermined, although two pathophysiological hypotheses have been proposed to explain sirenomelia: the vascular steal theory [21] and defective blastogenesis or failure of the development of ventral mesoderm [22]. These two pathophysiological hypotheses could be interrelated and may constitute a similar pathophysiological continuum. Abnormalities of blastogenesis would result in defects of the caudal vasculature of the embryo, leading to malformation of the targeted organs by ischemia and nutrient deficiency [23].

The association between caudal regression syndrome, VACTERL association and sirenomelia has been reported [24–27]. Single umbilical artery and renal anomalies are almost invariably present while gastrointestinal anomalies are variable and include a blind ending colon, rectal atresia, and imperforate anus. Vertebral defects, cardiac defects, esophageal atresia with tracheoesophageal fistula, radial agenesis, upper limb defects and anomalies of the central nervous system can also be found with sirenomelia. Although our case did not undergo an autopsy, some of the associated anomalies in the VACTERL spectrum were present: single umbilical artery, vertebral segmentation defects, anogenital anomalies, and upper limb defects. The clinical phenotypic overlap between caudal dysgenesis, VACTERL association, and sirenomelia in our patients is highlighted, lending support to the theory that these entities may be different manifestations of a single pathogenic process [24, 26].

In conclusion, we described the first case of prenatal sonographic images of sirenomelia with anencephaly and rachischisis totalis. Awareness of these rare associations will avoid missed diagnoses and facilitate prenatal counselling, thus highlighting the importance of a thorough ultrasound examination.
